# A MXene-Based Solvent-Free Nanofluid Lubricant for Friction and Wear Reduction

**DOI:** 10.3390/molecules31010051

**Published:** 2025-12-23

**Authors:** Wenfeng Zhu, Xuwu Luo, Junfeng Xie, Yaoming Zhang, Mifeng Zhao, Junhui Wei, Lei Li, Houbu Li, Peipei Li

**Affiliations:** 1 CNPC Tubular Goods Research Institute, Xi’an 710065, China; 2 State Key Laboratory of Oil and Gas Equipment, Xi’an 710065, China; 3 CNPC R & D Center for Ultra-Deep Complex Reservior Exploration and Development, Korla 841001, China; 4 Engineering Research Center for Ultra-Deep Complex Reservoir Exploration and Development, Korla 841001, China; 5 Xinjiang Key Laboratory of Ultra-Deep Oil and Gas, Korla 841001, China; 6 PetroChina Tarim Oilfield Company, Korla 841001, China; 7 School of Advanced Materials and Nanotechnology, Xidian University, Xi’an 710126, China

**Keywords:** solvent-free nanofluid, MXene, lubricant, surface engineering, friction and wear reduction

## Abstract

With the rapid advancements in industrial technology, the demand for high-performance lubrication has surpassed the capabilities of traditional solid or liquid lubricants. In this study, a novel MXene-based solvent-free lubricating nanofluid was developed through the surface functionalization of Ti_3_C_2_T_x_ MXene nanosheets. This innovative material combines the superior mechanical properties of solid Ti_3_C_2_T_x_ MXene nanosheets with the stable flow and rapid self-repairing capabilities of liquid lubricants. The successful synthesis of the MXene-based solvent-free nanofluid lubricant was confirmed through a series of characterization techniques, and it was demonstrated that this nanofluid maintained excellent flowability at room temperature. Subsequent tribological tests revealed that the friction coefficient and the wear performance of the MXene-based solvent-free nanofluid lubricant improved with increasing mass concentrations of Ti_3_C_2_T_x_ MXene nanosheets under consistently applied loads. These results indicate that the MXene-based solvent-free nanofluid lubricant significantly reduces friction and wear, showcasing its potential as a high-performance lubricant for industrial applications.

## 1. Introduction

The annual economic losses caused by friction and wear between metal components are immense. Friction is a primary contributor to part degradation, reduced equipment lifespan, and significant energy waste [1]. It is estimated that approximately one-third of the energy consumed in the global mining and transportation industries is due to overcoming friction [2,3]. Moreover, around 80% of mechanical failures are attributed to component wear [4,5]. Lubrication is a highly effective method for controlling friction and wear, helping to reduce energy loss and extend the operational life of equipment [6]. Lubricants minimize frictional wear between interacting surfaces and facilitate the smooth relative motion of solid components [7]. Lubricants are categorized into various types based on their physical or chemical properties, including solid lubricants (e.g., graphene) [8,9,10,11], gaseous lubricants (e.g., compressed air or other gases) [12,13], liquid lubricants (e.g., oils) [14,15,16], and semi-solid lubricants [17,18]. However, both solid and liquid lubricants have inherent limitations when used independently. Solid lubricants often suffer from poor heat dissipation, limited self-healing capabilities, and shorter lifespans [19,20,21,22], while liquid lubricants are highly dependent on ambient temperature and are prone to issues such as creep [23,24,25,26]. To address these challenges, integrating solid lubricants into liquid lubrication systems as filler components or leveraging chemical engineering techniques to transform solid-phase lubricants into liquid-phase systems can offer a promising solution. This approach combines the rapid self-repair, high mechanical strength, and stability of solid lubricants with the fluidity and adaptability of liquid lubricants, potentially resulting in a new generation of lubricants with enhanced load-bearing capacity and performance.

Fortunately, the combination of liquid fluidity and solid functionality through surface modification methods has provided a versatile platform for specific applications in recent decades [27,28,29,30]. For instance, Guo et al. [31] developed a solvent-free lubricant by modifying reduced graphene oxide with hyperbranched polyamine ester liquid using surface chemical engineering techniques. They observed significant improvements in the lubricant’s dispersibility and lubricity. Similarly, various surface modification strategies have been employed to synthesize porous liquids for applications in gas adsorption and separation. Li et al. [32] used imidazolium cationic-based Polymerized Ionic Liquids (PILs) to modify carbon networks, creating HCS@PILs. Mobility was achieved through an anion-exchange method by balancing the surface charge of HCS@PILs with Polyethylene Glycol (PEG). These strategies for preparing solvent-free nanofluids provide valuable insights and directions for the synthesis of advanced lubricants with superior performance in the future.

Two-dimensional (2D) nanomaterials have shown great potential as lubricants due to their exceptional physical and chemical properties [33,34]. For example, graphene [31,35,36] and hexagonal boron nitride (h-BN) [37] have been extensively studied as lubricants over the past decade [34,38,39,40,41,42]. Although the dispersion stability and chemical reaction mechanisms of these 2D materials as lubricants require further investigation [43], their excellent lubrication properties under extreme operating conditions (e.g., high temperature, high pressure, and high speed) have garnered increasing research interest and attention [44,45,46]. Ti_3_C_2_T_x_ MXene nanosheets, with their weakly bonded multilayer structure and self-lubricating properties, hold significant promise in the field of lubricants [47,48,49]. For instance, Rosenkranz et al. [50] investigated the effect of Ti_3_C_2_T_x_ MXene nanosheets on the friction and wear properties of highly loaded steel/steel dry sliding under varying contact pressures and relative humidity levels. They found that Ti_3_C_2_T_x_ MXene-coated samples exhibited a 2.3-fold reduction in friction and a 2.7-fold reduction in wear under moderate contact pressure and low relative humidity conditions. Additionally, the Ti_3_C_2_T_x_ MXene nanosheet layers are connected by relatively weak van der Waals forces, allowing the nanosheets to slip easily under friction, a unique property beneficial for lubrication [51,52]. Despite these advantages, Ti_3_C_2_T_x_ MXene still faces several challenges as a lubricant [53,54]. Firstly, its compatibility with different materials requires further exploration [55]. Secondly, its long-term stability needs improvement [54]. Many researchers have attempted to use Ti_3_C_2_T_x_ MXene nanosheets as lubricant additives to enhance friction performance by leveraging their excellent mechanical properties. However, Ti_3_C_2_T_x_ MXene nanosheets often exhibit poor dispersion in most base oils, limiting their effectiveness in improving friction performance [56,57,58]. Addressing these challenges will be crucial for unlocking the full potential of Ti_3_C_2_T_x_ MXene in lubrication applications.

**Scheme 1:**
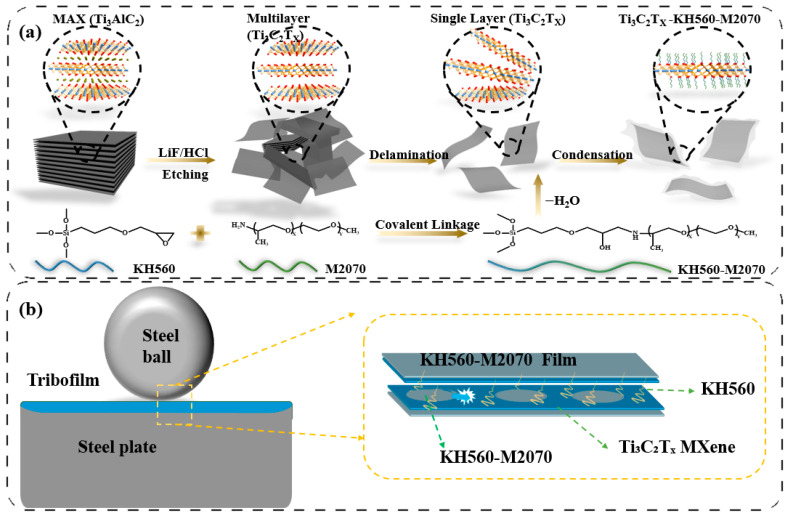
Diagram of (**a**) the preparation process and (**b**) the proposed lubrication mechanism of the MXene-based solvent-free nanofluid lubricant (Ti_3_C_2_T_x_-KH560-M2070).

In this study, a novel MXene-based solvent-free nanofluid lubricant was successfully designed and synthesized. As illustrated in Scheme 1a, the lubricant features a distinctive “core-neck-crown” architecture comprising three essential components: Ti_3_C_2_T_x_ MXene nanosheets serving as the structural core, the coupling agent γ-(2,3-epoxypropoxy)propyltrimethoxysilane (KH560) as an intermediate layer, and the amphiphilic polyether amine M2070 as the fluid canopy. This design concept shares similarities with solvent-free graphene lubricants, where functionalized graphene cores are typically grafted with organic coronas to achieve fluidity. However, unlike graphene-based systems that rely primarily on physical adsorption films and low interlayer shear, the MXene-based nanofluid exhibits a more sophisticated lubrication mechanism. As depicted in Scheme 1b, the Ti_3_C_2_T_x_ nanosheets not only facilitate interlayer sliding but also participate in the formation of a tribofilm enriched with Ti–O–C components, thereby enhancing interfacial adhesion and load-bearing capacity. Simultaneously, the KH560-M2070 polymer architecture enables rapid repair of damaged nanosheet structures. To evaluate the tribological performance, lubricants with MXene concentrations of 0.3 wt.%, 1.0 wt.%, and 2.5 wt.% were prepared and tested. CLSM analysis of the wear scars demonstrated that both the friction coefficient and wear decreased significantly with increasing MXene concentration under constant load. These findings confirm that the MXene-based nanofluid effectively integrates the self-lubricating characteristics of solid lubricants with the fluidity and self-healing capabilities of liquid lubricants, providing a robust and scalable strategy for developing high-performance lubricants suitable for demanding tribological conditions.

## 2. Result and Discussion

**Figure 1:**
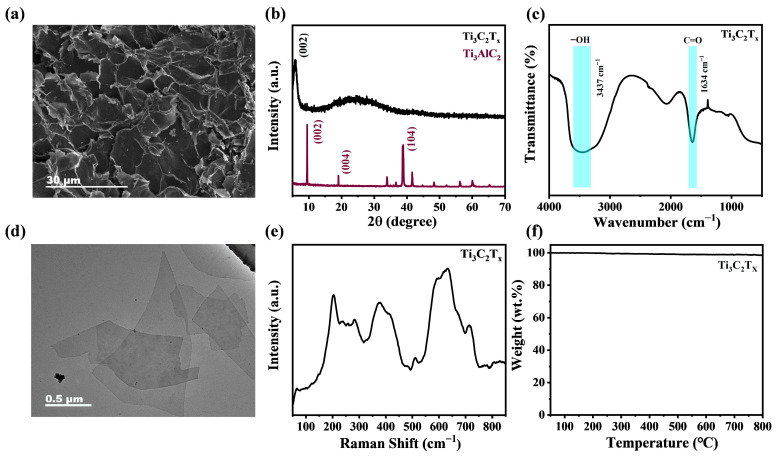
Characterization of the Ti_3_C_2_T_x_ MXene nanosheets. (**a**) SEM, (**b**) XRD pattern, (**c**) ATR-IR spectrum, (**d**) TEM, (**e**) Raman spectrum, and (**f**) TGA traces under N_2_ atmosphere at 5 °C/min, respectively.

Initially, the Ti_3_C_2_T_x_ MXene nanosheets obtained through etching exhibit a thin and lightweight sheet-like structure, as confirmed by SEM (Figure 1a) and TEM (Figure 1d) images. The XRD spectra of the precursor MAX-phase Ti_3_AlC_2_ powder before and after etching in the LiF/HCl mixture are shown in Figure 1b. After etching, the (002) diffraction peaks of Ti_3_AlC_2_ shift to a lower angle, and the strong diffraction peak at 39° disappears, indicating the selective removal of the Al atomic layer from the Ti_3_AlC_2_ structure [59,60]. ATR-IR spectroscopy (Figure 1c) reveals the presence of abundant hydroxyl groups on the surface of the Ti_3_C_2_T_x_ MXene nanosheets, which is crucial for subsequent covalent grafting. The Raman spectrum in Figure 1e shows that Ti_3_AlC_2_ exhibits features similar but not identical to those previously reported [61], likely due to variations in the type of MAX products received from different suppliers [62]. The TGA curves (Figure 1f) show no significant mass loss up to 800 °C, confirming that the Ti_3_C_2_T_x_ MXene nanosheets are free of volatile solvents. These results demonstrate the successful preparation of Ti_3_C_2_T_x_ MXene nanosheets that meet the experimental requirements, providing a solid foundation for further modification and covalent grafting of organic oligomers.

**Figure 2:**
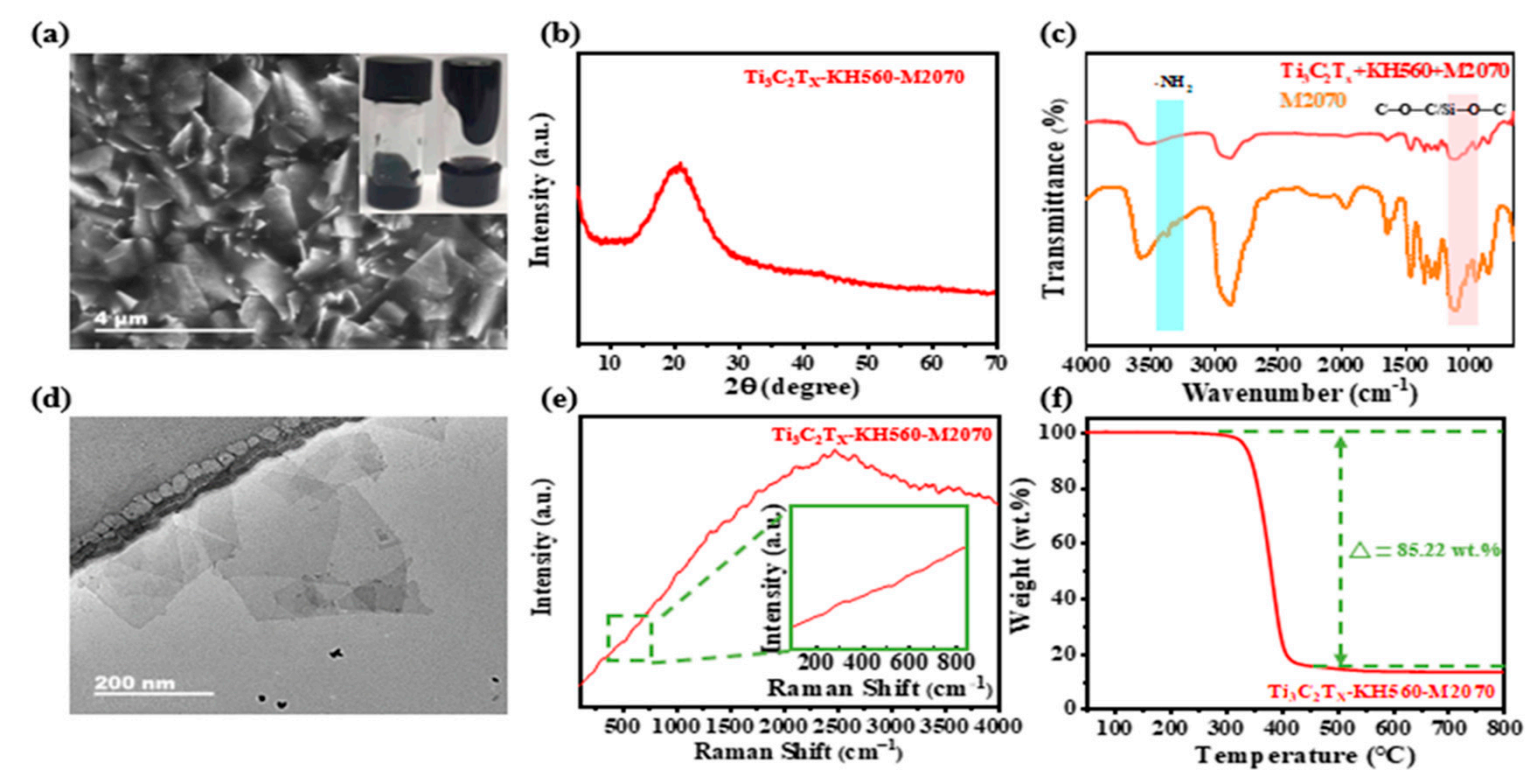
Characterization of the MXene-based solvent-free nanofluid (Ti_3_C_2_T_x_-KH560-M2070). (**a**) SEM (The inner picture shows the optical photograph of the MXene-based solvent-free nanofluid), (**b**) XRD pattern, (**c**) ATR-IR spectrum, (**d**) TEM, (**e**) Raman spectra (the green frame shows a partial enlargement), and (**f**) TGA traces under N_2_ atmosphere at 5 °C/min, respectively.

In Figure 2a, the prepared MXene-based solvent-free nanofluid is dispersed in a continuous medium and exhibits excellent flowability at room temperature. In contrast to the Ti_3_C_2_T_x_ MXene nanosheets, the XRD pattern of the MXene-based solvent-free nanofluid (Figure 2b) reveals a distinct peak at 20°, attributed to the organic oligomers grafted onto the surface of the nanosheets [63]. The ATR-IR analysis (Figure 2c) confirms the successful covalent functionalization of Ti_3_C_2_T_x_ MXene nanosheets. Significant spectral modifications are observed in the functionalized nanofluid compared to the pristine MXene. The emergence of characteristic C–H stretching vibrations at 2920 cm^−1^ and 2850 cm^−1^ provides clear evidence for the incorporation of organic constituents from KH560 and M2070. The spectral changes in the 3400 cm^−1^ region, coupled with the appearance of an amide-related absorption at 1640 cm^−1^, indicate the consumption of amine groups in M2070 through epoxy ring-opening with KH560. Additionally, the pronounced broad absorption between 1000 and 1100 cm^−1^, corresponding to Si–O–C and C–O–C stretching modes, verifies the covalent linkage established between the MXene sheets and the polymer canopy via the silane coupling agent. These collective spectroscopic findings substantiate the successful construction of the targeted “core–neck–crown” configuration through covalent surface modification. However, the characteristic peaks of the -NH_2_ group around 3400 cm^−1^ disappear, likely due to the epoxy ring-opening covalent reaction with KH560 [64]. This reaction forms a stable “core–neck–crown” architecture in which the KH560–M2070 polymer shell creates a compact protective layer around the MXene nanosheets. The shell effectively blocks thermal and oxidative attack, thereby significantly delaying the degradation of the MXene core. This protective effect is directly evidenced by the TEM image obtained after three months of storage (Figure 2d), where the nanosheets retain their structural integrity without observable oxidation. The suppression of MXene-related Raman features in Figure 2e also indirectly reflects the complete encapsulation by the organic coating. TGA (Figure 2f) provides quantitative support for this protection mechanism: the major mass loss of ~85.22 wt.% corresponds to decomposition of the polymer shell, which may consume local oxygen and generate a thin carbonaceous layer that further protects the MXene surface. Consequently, the sample maintains ~15 wt.% residual mass even at 800 °C, demonstrating the remarkable thermal stability endowed by the composite structure.

**Figure 3:**
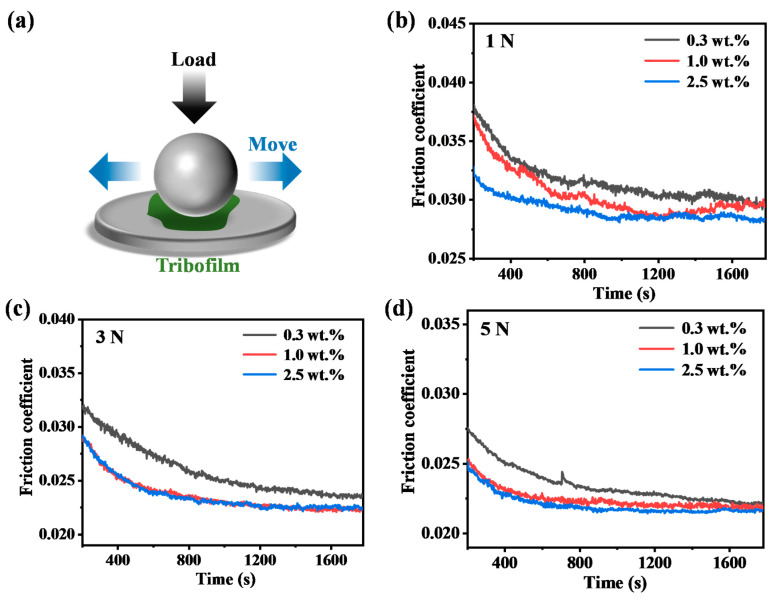
(**a**) Schematic working diagram of the tribometer and (**b**–**d**) The friction coefficients of the MXene-based solvent-free nanofluid lubricants with different concentrations of Ti_3_C_2_T_x_ MXene nanosheets and varied applied loads of 1 N, 3 N, and 5 N.

The prepared MXene-based solvent-free nanofluid demonstrates excellent stability and fluidity at room temperature, making it a promising candidate for use as a novel lubricant (i.e., MXene-based solvent-free nanofluid lubricant). The tribological properties of this lubricant were tested at three different concentrations of Ti_3_C_2_T_x_ MXene nanosheets: 0.3 wt.%, 1.0 wt.%, and 2.5 wt.%. Figure 3a provides a schematic of the tribometer setup used for friction testing. Figure 3b–d display the friction coefficients of the MXene-based solvent-free nanofluid lubricants with varying Ti_3_C_2_T_x_ MXene nanosheet concentrations (0.3 wt.%, 1.0 wt.%, and 2.5 wt.%) under applied loads of 1 N, 3 N, and 5 N. A clear trend is observed: the stable friction coefficients of the MXene-based solvent-free nanofluid lubricants decrease as the concentration of Ti_3_C_2_T_x_ MXene nanosheets increases, regardless of the applied load. For example, under a 1 N load, the stable friction coefficients are approximately 0.032 (0.3 wt.%), 0.030 (1.0 wt.%), and 0.028 (2.5 wt.%). This trend of decreasing friction coefficients with increasing nanosheet concentration is consistent across all tested loads (1 N, 3 N, and 5 N). Furthermore, the stable friction coefficients also decrease as the applied load increases. The lowest friction coefficient, approximately 0.022, is achieved with the 2.5 wt.% Ti_3_C_2_T_x_ MXene nanosheets concentration under a 5 N load, as illustrated in Figure 3d. These results highlight the effectiveness of the MXene-based solvent-free nanofluid lubricant in reducing friction under varying conditions.

**Figure 4:**
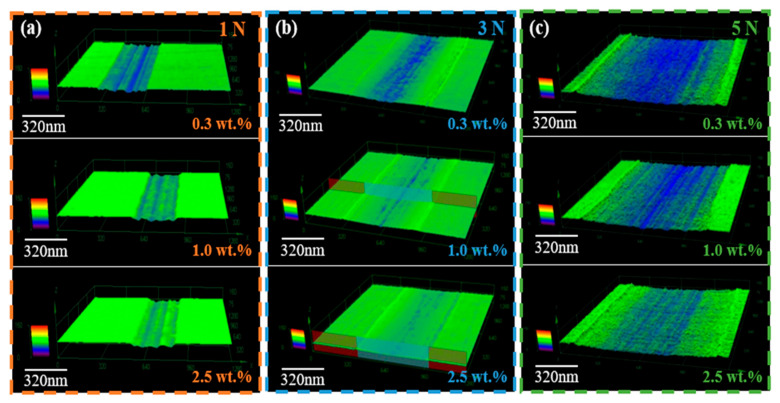
CLSM was used to characterize the wear morphology of the MXene-based solvent-free nanofluid lubricants with different concentrations of Ti_3_C_2_T_x_ MXene nanosheets. The applied loads are (**a**) 1 N, (**b**) 3 N, and (**c**) 5 N, respectively.

The wear morphology of steel plates lubricated with the MXene-based solvent-free nanofluid under varying applied loads was characterized using Confocal Laser Scanning Microscopy (CLSM). As illustrated in Figure 4a–c, under loads of 1 N, 3 N, and 5 N, the wear scars consistently became shallower and narrower with increasing mass concentration of Ti_3_C_2_T_x_ MXene nanosheets, indicating a clear correlation between nanosheet concentration and enhanced anti-wear performance. This improvement stems from the synergistic behavior of the nanofluid’s multi-component architecture: the Ti_3_C_2_T_x_ MXene nanosheets act as a solid lubricant by promoting interlayer shear and in situ formation of a protective tribofilm, while the fluid polyether amine canopy (M2070) ensures low shear stress and rapid replenishment of wear zones. The KH560 coupling agent covalently links the MXene and polymer, reinforcing the structural integrity and enabling a robust, adaptive, and self-repairing lubricating system. Together, these features allow the nanofluid to spread uniformly, maintain a continuous lubricating film, and rapidly recover damaged areas, thereby stabilizing the friction coefficient and combining the key advantages of solid and liquid lubricants for effective friction and wear reduction.

## 3. Experimental Section

### 3.1. Materials

Layered ternary carbide (Ti_3_AlC_2_) MAX phase powder with a 200-mesh size was purchased from Forsman Technology Co., Ltd. (Beijing, China). The organosilane coupling agent γ-(2,3-epoxypropoxy) propyltrimethoxysilane (KH560, AR, 98%) and lithium fluoride (LiF, ≥98%) were obtained from Aladdin Reagent Co., Ltd. (Shanghai, China). Polyether amine M2070 was supplied by Dalian Liansheng Trading Co., Ltd. (Dalian, China). Concentrated hydrochloric acid was purchased from Sinopharm Chemical Reagent Co., Ltd. (Shanghai, China). All chemicals were used as received without further purification.

### 3.2. Preparation of Ti_3_C_2_T_x_ MXene Nanosheets

The conventional two-step method for obtaining Ti_3_C_2_T_x_ MXene nanosheets, which was adopted in the study reported here, involves etching the aluminum atomic layer from the MAX phase using HF acid. As illustrated in Scheme 1, 15 mL of 12 mol/L HCl was first mixed with 5 mL of deionized water to prepare 20 mL of a 9 mol/L HCl solution. Next, 1.6 g of LiF was slowly added to the diluted HCl solution and stirred for 10 min using a Polytetrafluoroethylene (PTFE) magnetic stirrer. Afterward, 1 g of Ti_3_AlC_2_ powder was gradually introduced into the HCl/LiF mixture. The mixture was allowed to react at 35 °C for 24 h under continuous stirring. Once the reaction was complete, the mixture was centrifuged with deionized water at 4000 rpm until the supernatant reached a neutral pH. The resulting product was then sonicated for 1 h under nitrogen protection to exfoliate the MXene layers. Finally, the mixture was centrifuged at 3500 rpm for 30 min to isolate few-layer or monolayer Ti_3_C_2_T_x_ MXene nanosheets in an aqueous solution. This solution was subsequently used as the base material for preparing the nanofluids in this study.

### 3.3. Synthesis of the MXene-Based Solvent-Free Nanofluid Lubricant (Ti_3_C_2_T_x_-KH560-M2070)

In the preparation process, 10 g of M2070 and an equimolar amount of KH560 were dissolved in 100 mL of methanol. The solution was then subjected to condensation and reflux at a constant temperature of 45 °C for 12 h. Following this, an aqueous solution of Ti_3_C_2_T_x_ MXene (50 mg/mL) was added to the KH560-M2070 methanol solution, and the mixture was stirred at room temperature for an additional 12 h. The resulting dispersion was transferred into a dialysis bag with a molecular weight cut-off of 5000 and dialyzed for 2 days, with the deionized water being replaced 4–6 times during this period. After dialysis, the excess water in the dispersion was removed using rotary evaporation. This process yielded the final product: a MXene-based solvent-free nanofluid lubricant (Ti_3_C_2_T_x_-KH560-M2070).

### 3.4. Characterization

The attenuated total reflection infrared (ATR-IR) spectra were acquired using a Nicolet iS50 intelligent ATR-IR spectrometer (Thermo Scientific, Waltham, MA, USA) with a spectral range of 500–4000 cm^−1^. Scanning electron microscopy (SEM) images were captured at an accelerating voltage of 5 kV. For transmission electron microscopy (TEM) imaging, a sample dispersion (5 mg/mL) was drop-cast onto carbon-coated copper grids and allowed to dry naturally at room temperature before imaging at 120 kV. Thermogravimetric analysis (TGA) was conducted using a Q50 TA instrument under a nitrogen atmosphere, with a heating rate of 5 °C/min. Raman spectroscopy measurements of Ti_3_C_2_T_x_ MXene nanosheets and the MXene-based solvent-free nanofluid lubricant were performed using a 532 nm laser (Renishaw inVia, Pliezhausen, Germany). Friction and wear tests were conducted using a Tribometer (Center for Tribology, Campbell, CA, USA), while wear scar characterization was performed using a Confocal Laser Scanning Microscope (CLSM, Olympus Corporation, Tokyo, Japan). The wear tests employed a ball-on-disk configuration (CSM tribometer (TRB-III), Jinan Zhongwei Casting and Forging Grinding Ball Co., Ltd., Jinan, China), where the friction and wear behavior of a steel plate coated with the MXene-based solvent-free nanofluid lubricant was evaluated against a steel ball (AISI 52100 steel, 6 mm diameter, Ra ≈ 20 nm, Jinan Zhongwei Casting and Forging Grinding Ball Co., Ltd., Jinan, China) in a linear reciprocating motion. Prior to testing, the 304 stainless steel plate was polished, cleaned, and coated with the MXene-based solvent-free nanofluid lubricant. The tests were conducted under applied loads of 1 N, 3 N, and 5 N at a constant sliding speed of 60 mm/s, a wear track length of 10 mm, and a test duration of 30 min. All tribological measurements were performed under ambient conditions of 25 ± 2 °C and 45 ± 5% relative humidity. After the tests, the morphology of the wear scars on the 304 stainless steel plate was analyzed using CLSM.

## 4. Conclusions

In the study reported here, a MXene-based solvent-free nanofluid was successfully developed through the surface functionalization of Ti_3_C_2_T_x_ MXene nanosheets using the coupling agent γ-(2,3-epoxypropoxy) propyltrimethoxysilane (KH560) and the amphiphilic long-chain liquid polyether amine M2070, constructing a distinctive “core–neck–crown” architecture. The resulting nanofluid exhibits excellent stability and fluidity at room temperature, making it suitable for use as a novel lubricant. Subsequent tribological characterization revealed that both the stable friction coefficients and wear decrease significantly with increasing mass fraction of Ti_3_C_2_T_x_ MXene nanosheets under varying applied loads, indicating outstanding friction-reduction and anti-wear performance. More importantly, integrated structural and tribological analysis reveals a solid–liquid synergistic lubrication mechanism during sliding: the MXene nanosheets, serving as the solid lubricating core, form a protective tribofilm with low shear strength due to their facile interlayer slip, while the outer M2070 liquid polymer provides fluid lubrication and flexible cushioning, enabling rapid replenishment of worn areas. Covalently linked by KH560, these two components form a stable hybrid, resulting in a continuous, flexible, and self-repairing composite lubrication film at the friction interface. This solid–liquid synergy is identified as the fundamental reason for the superior tribological performance.

The findings of this work demonstrate that the MXene-based solvent-free nanofluid lubricant not only effectively reduces friction and wear but also overcomes the drawbacks of conventional MXene additives in oil-based systems, such as poor stability and agglomeration, while exhibiting the potential to combine the advantages of both solid and liquid lubricants. This study thereby offers a viable route for developing high-performance lubricating materials capable of operating under demanding industrial conditions.

## Data Availability

The original contributions presented in this study are included in the article. Further inquiries can be directed to the corresponding authors.
